# Long-Term Overgrazing-Induced Memory Decreases Photosynthesis of Clonal Offspring in a Perennial Grassland Plant

**DOI:** 10.3389/fpls.2017.00419

**Published:** 2017-04-24

**Authors:** Weibo Ren, Ningning Hu, Xiangyang Hou, Jize Zhang, Huiqin Guo, Zhiying Liu, Lingqi Kong, Zinian Wu, Hui Wang, Xiliang Li

**Affiliations:** ^1^Key Laboratory of Grassland Ecology and Restoration of Ministry of Agriculture, National Forage Improvement Center, Institute of Grassland Research, Chinese Academy of Agricultural SciencesHohhot, China; ^2^College of Life Sciences, Inner Mongolia Agricultural UniversityHohhot, China; ^3^College of Ecology and Environment, Inner Mongolia UniversityHohhot, China

**Keywords:** phenotypic plasticity, transgenerational effect, photosynthesis, grassland, livestock grazing

## Abstract

Previous studies of transgenerational plasticity have demonstrated that long-term overgrazing experienced by *Leymus chinensis*, an ecologically dominant, rhizomatous grass species in eastern Eurasian temperate grassland, significantly affects its clonal growth in subsequent generations. However, there is a dearth of information on the reasons underlying this overgrazing-induced memory effect in plant morphological plasticity. We characterized the relationship between a dwarf phenotype and photosynthesis function decline of *L. chinensis* from the perspective of leaf photosynthesis by using both field measurement and rhizome buds culture cultivated in a greenhouse. Leaf photosynthetic functions (net photosynthetic rate, stomatal conductance, intercellular carbon dioxide concentration, and transpiration rate) were significantly decreased in smaller *L. chinensis* individuals that were induced to have a dwarf phenotype by being heavily grazed in the field. This decreased photosynthetic function was maintained a generation after greenhouse tests in which grazing was excluded. Both the response of *L. chinensis* morphological traits and photosynthetic functions in greenhouse were deceased relative to those in the field experiment. Further, there were significant decreases in leaf chlorophyll content and Rubisco enzyme activities of leaves between bud-cultured dwarf and non-dwarf *L. chinensis* in the greenhouse. Moreover, gene expression patterns showed that the bud-cultured dwarf *L. chinensis* significantly down-regulated (by 1.86- to 5.33-fold) a series of key genes that regulate photosynthetic efficiency, stomata opening, and chloroplast development compared with the non-dwarf *L. chinensis*. This is among the first studies revealing a linkage between long-term overgrazing affecting the transgenerational morphological plasticity of clonal plants and physiologically adaptive photosynthesis function. Overall, clonal transgenerational effects in *L. chinensis* phenotypic traits heavily involve photosynthetic plasticity.

## Introduction

Ecologists have long been interested in phenotypic plasticity and its role in many ecological and evolutionary biological processes (Turcotte and Levine, 2016). The widely accepted definition of phenotypic plasticity is the performance of multiple phenotypes from a single plant genotype, in a manner dependent on environmental stimuli (Anderson et al., 2012). Theory predicts that greater phenotypic plasticity can result from the morphological, physiological, and phenological adaptive capacities in which plants can adjust their growing performances under various environmental variations (Kooke et al., 2015; Li X. et al., 2016).

Several studies have also reported that phenotypic changes could be observed over several generations in some cases (Mcintyre and Strauss, 2014). This ecological phenomenon is defined as transgenerational plasticity (Agrawal, 2002; Vu et al., 2015). Through this process, variation in environmental factors in the maternal generation, such as light, nutrients, water, pathogenic bacteria, and livestock grazing, influence the growth performance, life history, and biomass allocation of the progeny plants (Galloway and Etterson, 2007; Herman and Sultan, 2016). Recent progress in transgenerational plasticity research has experimentally demonstrated that local phenotypic adjustments under multiple stresses can transfer to the subsequent generation asexually under both uniform and contrasting environments in clonal plants (Latzel and Klimesova, 2010a; Gonzalez et al., 2016).

Similarly, natural grassland plants in are constantly subjected to various biotic and abiotic stresses as their habitats change seasonally and over time (Couso and Fernandez, 2012). A growing body of experimental evidence suggests that grassland plants show rapid phenotypic plasticity when exposed to such biotic and abiotic stresses (Li et al., 2015a). For example, Couso and Fernandez (2012) reported that the most tolerant species had the highest morphological plasticity in an underlying trait in three Patagonian steppe grasses. In addition, previous studies have shown that light availability experienced by plant significantly influences the ability of following generations to respond to shade (Heger, 2016).

Typical steppe grasslands in Inner Mongolia, China, which are mainly dominated by *Leymus chinensis*, cover a large area of the eastern Eurasian temperate grassland (Schönbach et al., 2009). *L. chinensis* is a native, perennial, rhizomatous grass with high palatability and forage value; it is one of the most preferred species for large herbivores (Liu and Han, 2008; Huang et al., 2015). Therefore, it sustains higher relative levels of grazing by large herbivorous (Wang et al., 2010). Given the low genetic variation of clonal plants across a wide range of habitats, phenotypic plasticity can serve as the primary adaptive strategy, thereby allowing them to thrive in many grasslands (Aalto et al., 2014; Latzel et al., 2016; Liu Y. et al., 2016). Using large-scale field sampling and controlled watering experiments, Liu Y. et al. (2016) found that phenotypic plasticity in water use efficiency was more important than local adaptation in allowing the clonal *L. chinensis* to occupy a wide range of grassland habitats.

Understanding the biological processes of plant phenotypic plasticity in response to livestock grazing can provide insight into the process of grazing-induced changes in grassland ecosystem functioning. Our previous research on clonal transgenerational plasticity in grassland plant indicated that the significant response of morphological plasticity in *L. chinensis* with and without long-term overgrazing was maintained in the clonal offspring in a hydroponic experiment designed to remove the maternal environment (Li et al., 2015b). Importantly, leaf photosynthesis is the primary factor determining the carbohydrate sources of plant growth and development (Ellsworth et al., 2004). A growing body of empirical evidence from grazing ecosystems suggests that overgrazing by livestock may dramatically restrict leaf photosynthetic capacity in field conditions (Shen et al., 2013). However, there is a dearth of information on the linkages between plant morphological plasticity and leaf photosynthesis plasticity induced by livestock grazing.

In the present study, we used a long-term grazing and grazing-exclusion field experiment to evaluate the effects of grazing on plant morphological and photosynthesis plasticity in *L. chinensis*. In particular, we investigated the memory effect of photosynthesis function by culturing rhizome buds to examine the underlying reasons in physiology, ultrastructure, and gene expression. We addressed three main research questions. (i) Is there an effect of grazing on plant performance of rhizome-cultured *L. chinensis*? (ii) What is the relationship between the memory effect of plant morphological plasticity and changes in photosynthetic characteristics? (iii) How is the transgenerational memory effect in photosynthetic plasticity of *L. chinensis* regulated by physiological and molecular pathways?

## Materials and Methods

### Field Site and Plants

**FIGURE 1:**
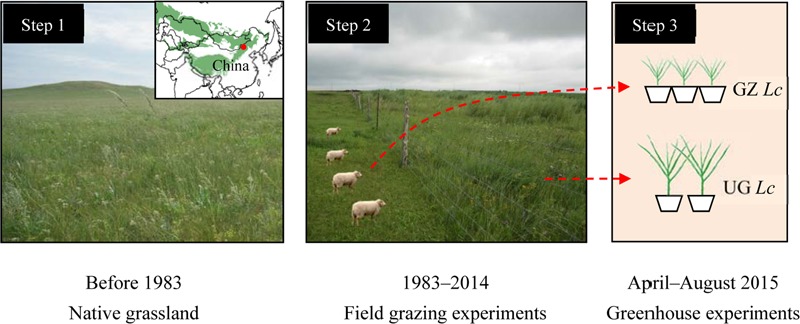
**Scheme and schedule of the experimental design.** The green area in the inlaid partial map of Asia shows the distribution of natural grassland, and the red dot represents our study area. GZ *Lc*, clone offspring of *Leymus chinensis* from long-term overgrazed grassland; UG *Lc*, clone offspring of *L. chinensis* from ungrazed grassland.

We conducted our phenotypic plasticity field experiment at the Inner Mongolia Grassland Ecosystem Research Station (IMGERS, 43°38′ N, 116°42′ E, **Figure 1**). The study site is located in the Xilin River catchment, China at an altitude of approximately 1,200 m above sea level. The semiarid continental climate is characterized by a mean annual (1982–2008) temperature of 0.7°C and a mean annual precipitation of 335 mm (Supplementary Figure S1). Owing to inter-annual variability, the coefficient of variation of precipitation is 22% (Li et al., 2015a). At our field site, the highest temperature coincides with the maximum precipitation in June, July, and August each year. For perennial plants, the growing season lasts for approximately 150 days from April/May to September/October, whereas annual plants germinate later, typically in July following the period of highest rainfall. The perennial rhizome grass *L. chinensis* and bunch grass *Stipa grandis* dominate the typical steppe communities of our study site (Li X. et al., 2016). And calcic kastanozem and calcic chernozems are the major soil types of this area. In this study, we selected *L. chinensis* as the model species for examining phenotypic plasticity in response to long-term overgrazing in the experiments. *L. chinensis*, dominates the Inner Mongolia grasslands, is widespread from the southern Chinese Loess plateau to the northern Russian Baikal and from the Sanjiang plain of eastern China to Ulan Bator in Mongolia (Liu Y. et al., 2016). In addition, *L. chinensis* is a rhizomatous native perennial plant with good palatability and high forage value. It is highly adaptable across grazed grassland ecosystems (Li et al., 2015b).

### Field Experiment

The experimental design employed in the current study was previously described by Li et al. (2015b). Nearby long-term freely grazing and grazing-exclusion (ungrazed) plots were established in the study area. The grazing exclusion plot has been protected from grazers by a fence since 1983 by IMGERS for the purpose of long-term ecological observation and research. The grazing plot, of more than 200 ha in area, was situated adjacent to the grazing-exclusion plot and grazed by more than 600 sheep and goats year round over the last several decades (>30 years; Li et al., 2015b). In the two groups plots (i.e., grazed and ungrazed), three secondary plots were set, respectively. Five 1 m × 1 m quadrats were randomly selected in each of the secondary plots. On the grazed plots, temporary movable ex-closure cages were set up at each sampling quadrat prior to grazing before the growing season in early April, 2014. The field sampling was carried out during 10–20 August 2014, corresponding to the annual peak-standing biomass (Supplementary Figure S1).

### Greenhouse Experiment

Soil culture methods were employed in our greenhouse experiment. Before the start of the *L. chinensis* growing season, rhizome buds that had been at the same initial development state in the two experimental groups (grazed and ungrazed) were collected, respectively, in April 2015. In the laboratory, samples were clipped into 2-cm lengths, and the rhizome buds of *L. chinensis* were cultivated in flowerpots, 30 cm in diameter and 25 cm high, in the same resource environment to remove the impact of nutrition, water, illumination, etc., in long-term overgrazing plots. Vermiculite and nutritional soil (1:2 ratio) were well mixing in each flowerpot, and each was planted with five rhizome buds. Six flowerpots planted with *L. chinensis* rhizomes were cultivated for each treatment. In total, these 12 flowerpots were randomly arranged in the greenhouse (at 25°C under light and 15°C under dark cycles with natural light and regular watering), which was located at Institute of Grassland Research, Chinese Academy of Agricultural Sciences, Hohhot, Inner Mongolia, China.

### Measurements

#### Morphological Traits

We measured morphological, physiological, and molecular properties of *L. chinensis* leaves at the rapid and peaking growth period in the field (early August) and laboratory (45 days after seedling establishment). The phenotypic traits we measured in *L. chinensis* were plant height, leaf length, leaf width, stem length, and stem diameter. The measuring methods employed in the current study were described in a previous publication (Li et al., 2015a). Both in the field and greenhouse experiment, leaf length and leaf width were measured from the second leaf from the bottom of each plant, which is the youngest mature leaf of each individual. The stem diameter was measured at the second internode from the bottom of *L. chinensis* stems. First, in the field experiment, three *L. chinensis* individuals were selected randomly in each 1 m × 1 m quadrat. Phenotypic traits of *L. chinensis* were measured in a darkened laboratory room after the whole aboveground portion of plant individuals were clipped completely in the field. As in our field experiment, after 45 days of cultivation, the same phenotypic traits of the mature *L. chinensis* plants were measured from all 12 of the flowerpots in our greenhouse testing using the same methods and indexes. These measurements were taken from three *L. chinensis* selected randomly from each flowerpot.

#### Photosynthetic Capability

At the same time morphological traits were measured, photosynthetic capability was measured from the second leaf from the bottom of each *L. chinensis* plant both in the field and greenhouse experiment. Net photosynthetic rate (*P*_N_), stomatal conductance (*g*s), intercellular carbon dioxide concentration (*C*_i_), and transpiration rate (*E*) were determined using a portable open flow gas exchange system LI–6400XT (LI-COR, Lincoln, NE, USA) at 2-h intervals from 9:00 to 11:00 h in the field. Intrinsic water use efficiency (*WUE*) was calculated as *PN/E*. Photosynthetically active radiation (*PAR*) was 1000 ± 10 μmol m^-2^ s^-1^, CO_2_ concentration was 350 ± 3 ppm, and leaf temperature was 27.0 ± 1.1°C (Reich et al., 2003; Li et al., 2014).

#### Chlorophyll Content

The content levels of chlorophyll a and b were measured using UV spectrophotometry (Laureau et al., 2013). Leaf tissues (5 g) were placed into test tubes, extracted with 30 ml of DMSO, and incubated overnight at 70°C. Chlorophyll contents were calculated at 665 and 649 nm by a spectrophotometer. Chlorophyll contents were expressed as mg/g fresh weight.

#### Chloroplast Ultrastructure

For the ultrastructural analysis, we examined samples of *L. chinensis* leaves that were grazed or ungrazed using a rhizome bud culture method in the greenhouse, with samples from corresponding sampling dates measured for each morphological and physiological measurement. For each treatment we used three *L. chinensis* leaves. The samples were fixed in 2.5% glutaraldehyde solution in 0.1 M phosphate buffer (pH 7.3) for 24 h at 5°C, post-fixed with 1% osmium tetroxide in the same buffer for 1 h at 25°C, dehydrated through an acetone series, and embedded in Araldite resin. Ultra-thin sections were obtained with a DiATOME diamond knife (DiATOME, Hatfield, PA, USA) and post-stained with uranyl acetate and lead citrate (Reynolds, 1963). The material was examined in an FEI Tecnai^TM^ transmission electron microscope (FEI, Hillsboro, OR, USA) at 80 kV.

#### Rubisco Enzyme Activity

In our measurement, 0.5 g leaf sample was rapidly ground in a 15-mL tube with liquid N_2_. Then spectrophotometry and Rubisco Activase Assay Kit was used to determine the enzyme activity according to a procedure in literature (Bernacchi et al., 2005; Li X.-J. et al., 2016).

#### Gene Expression

At the same time, leaves samples of *L. chinensis* were collected from the two greenhouse experimental groups (clonal offspring grazing and ungrazing *L. chinensis*). Leaf material that was snap frozen and stored at -80°C was after being ground to a fine powder in liquid N_2_ with a mortar and pestle (Liu Z.-Y. et al., 2016). The potential roles of the 12 studied genes, which is previously identified as having a role in plant photosynthesis (Boonman et al., 2009), has been preliminarily confirmed by our transcriptome data (Supplementary Table S1).

Total RNA was extracted from the *L. chinensis* leaves with TRIzol Reagent (Invitrogen, Carlsbad, CA, USA). Total RNA from each sample was reverse transcribed using Prime Script^TM^ RT reagent kit with gDNA Eraser (Perfect Real Time; Clontech, Mountain View, CA, USA) and stored at -20°C (Supplementary Figure S2). Primers used for gene amplification are listed in Supplementary Table S1. Primers were designed for a reference gene Actin1 (GenBank accession number, HM623326.1). Primers were designed using the online primer design software PerlPrimer v.1.1.21.

The method of quantitative real-time PCR has been described by previous studies (Livak and Schmittgen, 2001; Liu Z.-Y. et al., 2016). Therefore, we briefly describe the experimental process in this paper. Quantitative real-time PCR was carried out in an iQ5 Optical System (Bio-Rad, Hercules, CA, USA) using the SYBR Premix Ex Taq Kit (TaKaRa Biomedical, Shiga, Japan; Liu Z.-Y. et al., 2016). The relative expression level was calculated using the comparative C(t) method (which notes the PCR cycle at which the fluorescent signal of the reporter dye crosses an arbitrary threshold; Supplementary Figure S3; Peng et al., 2011; Liu Z.-Y. et al., 2016).

### Statistical Analysis

Before all of the statistical analyses were conducted, the phenotypic traits were averaged from three *L. chinensis* individuals in one quadrat in the field experiment or individual flowerpot in the greenhouse experiment (Li et al., 2015a; Li X. et al., 2016). The relative concentration of each gene of interest from the qRT-PCR analysis was log-transformed before analysis. Significant differences in each index, including plant phenotypic traits, photosynthetic characteristics, chlorophyll content, Rubisco enzymatic activity, and gene expression quantities, between the ungrazed and grazed groups were evaluated by Student’s *t*-test. The responses of phenotypic traits and photosynthetic characteristics were calculated by Ln(control/grazed; (Zheng et al., 2012). Linear correlations in this research were analyzed by the Pearson method. The non-linear relationship between *g*s and *P*_N_ was fitted with an equation describing exponential rise to a maximum (*y* = *y_0_* + *a* (1 - *e^-bx^*)). All statistical analyses were performed to determine the significance of treatment means at *P* < 0.05 and *P* < 0.01 significance levels using SPSS 19.0 statistical software (SPSS, Inc., Chicago, IL, USA).

## Results

**FIGURE 2:**
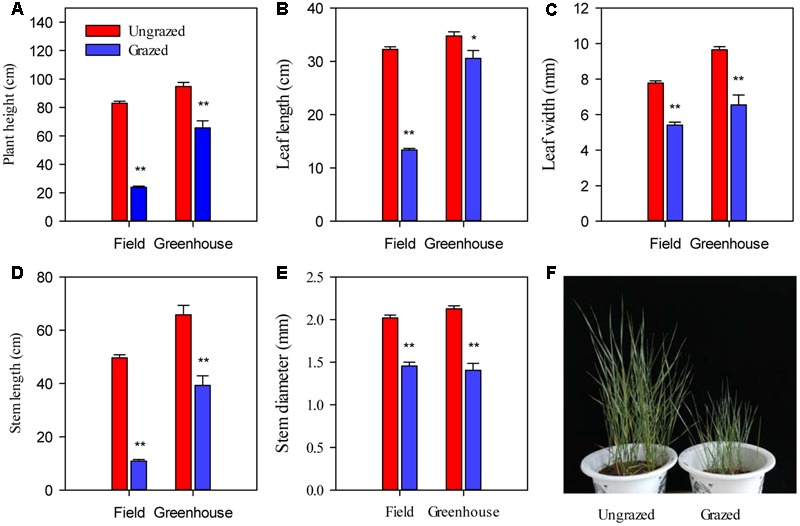
**Differences in plant growth and various morphological traits in *Leymus chinensis* in response to previous generation grazing in field and greenhouse conditions. (A–E)** The morphological traits indicated on the *x*-axis. **(F)** Photographs of bud culture *L. chinensis* in the greenhouse. The cultured *L. chinensis* buds were collected from the field, specifically the long-term overgrazing and grazing exclusion grasslands. Symbols: ^∗∗^*P* < 0.01; ^∗^*P* < 0.05.

Long-term overgrazing significantly decreased the plant size of *L. chinensis* individuals, including plant height, leaf length, leaf width, stem length, and stem diameter (*P* < 0.01, **Figure 2**). Moreover, we observed significant responses in morphological plasticity between the grazed and ungrazed treatments were maintained in the greenhouse experiments that were designed to remove environmental variability by the bud culture method (*P* < 0.05, **Figure 2**).

**FIGURE 3:**
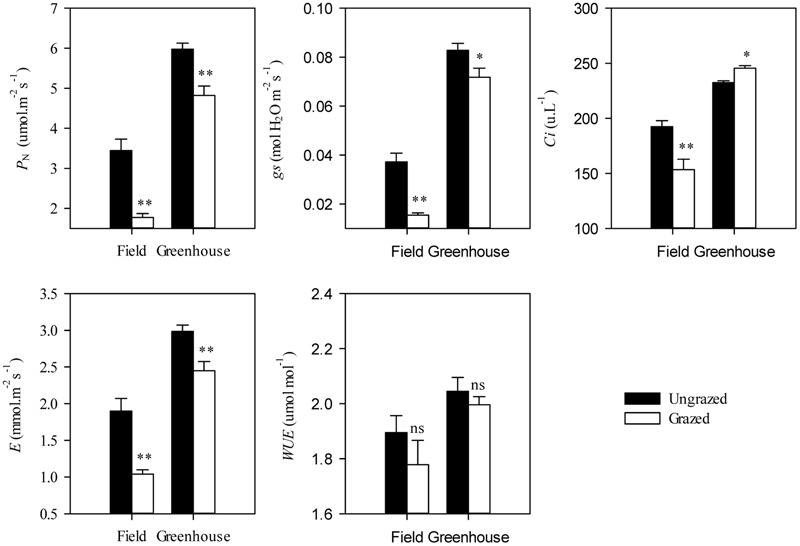
**Effects of grazing on leaf photosynthetic traits in *Leymus chinensis* under field and greenhouse experiments.** *P*_N_, Net photosynthetic rate; *g*s, Stomatal conductance; *C*i, Intercellular carbon dioxide concentration; E, Transpiration rate; and WUE, water use efficiency. Symbols: ^∗∗^*P* < 0.01; ^∗^*P* < 0.05; ns, *P* > 0.05.

**FIGURE 4:**
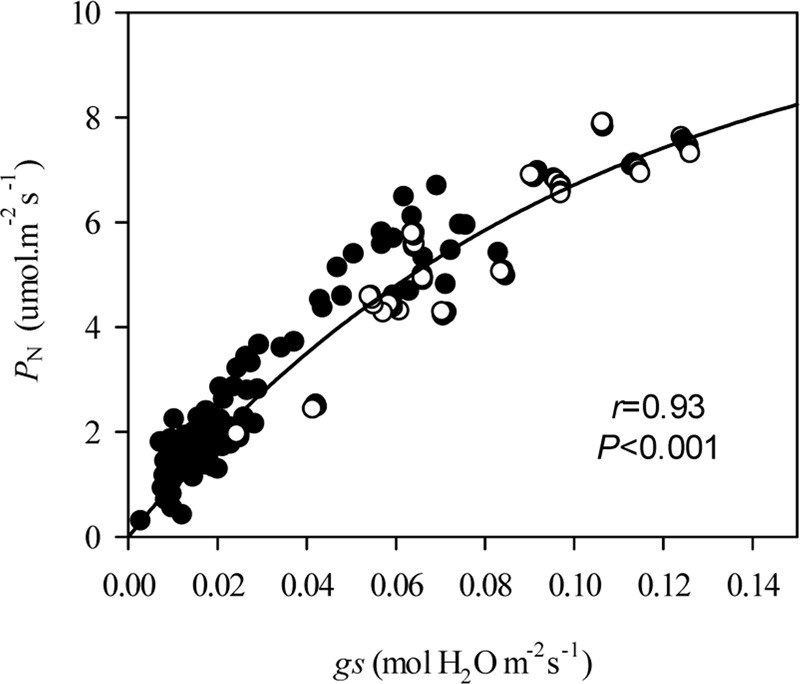
**Non-linear relationships of plasticity in stomatal conductance on net photosynthetic rate of *Leymus chinensis* leaves in different treatments.** The relationships between *g*s and *P*_N_ were fitted with an equation describing exponential rise to a maximum [(*y* = *y_0_* + *a* (1 - *e^-bx^*)), *r* = 0.93, *P* < 0.001]. *P*_N_, Net photosynthetic rate; *g*s, Stomatal conductance; •, field; and ∘, soil culture experiment in greenhouse.

Under grazing, photosynthetic functions, such as *P*_N_, *g*s, *C*i, and *E*, were significantly decreased in a manner that corresponded with the dwarf phenotype of *L. chinensis* individuals in field conditions (*P* < 0.01). In contrast, *WUE* was not affected by grazing (*P* > 0.05, **Figure 2**). This decreased photosynthetic function was maintained after one generation of grazing exclusion in greenhouse tests (**Figure 3**). Moreover, *P*_N_ values were significantly correlated with *C*i and *E* not only in the field but also in greenhouse tests (**Table 1**) and especially correlated with *g*s (**Figure 4**).

**Table 1:** Correlations among photosynthetic characteristics of *Leymus chinensis* leaf in field and greenhouse conditions.

Photosynthetic characteristics	Field		Common garden	
	*r*	significance	*r*	Significance
*P*_N_ *– g*s	0.95	^∗∗^	0.92	^∗∗^
*P*_N_ *– C*i	0.16	ns	*-*0.20	^∗^
*P*_N_ *– E*	0.95	^∗∗^	0.90	^∗∗^
*P*_N_ *– WUE*	0.08	ns	0.13	ns
*g*s *– C*i	0.39	^∗∗^	0.19	^∗^
*g*s *– E*	0.99	^∗∗^	0.90	^∗∗^
*g*s *– WUE*	*-*0.15	ns	*-*0.06	ns
*C*i *– E*	0.37	^∗∗^	*-*0.06	ns
*C*i *– WUE*	*-*0.84	^∗∗^	*-*0.37	^∗∗^
*E – WUE*	*-*0.19	ns	*-*0.30	^∗∗^

*P_*N*_, Net photosynthetic rate; *g*s, Stomatal conductance; *C*i, Intercellular carbon dioxide concentration; E, Transpiration rate; and WUE, water use efficiency. ^∗∗^*P* < 0.01; ^∗^*P* < 0.05; ns, *P* > 0.05.*

**FIGURE 5:**
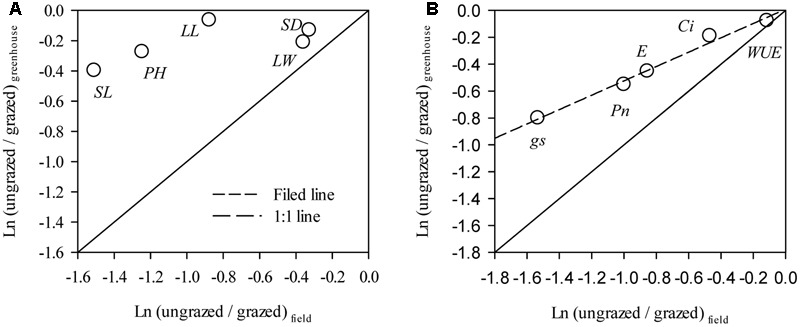
**Comparisons between response in *Leymus chinensis* (A)** morphological traits and **(B)** leaf photosynthetic traits to overgrazing in field and greenhouse experiments. The relationships for morphological traits (*R*^2^ = 0.47, *P* = 0.20) and leaf photosynthetic traits (*R*^2^ = 0.98, *P* < 0.001) between the responds ratios of field and greenhouse experiments were fitted by linear regression. PH, Plant height; LL, Leaf length; LW, Leaf width; SL, Stem length; SD, stem diameter; *P*_N_, Net photosynthetic rate; *g*s, Stomatal conductance; *C*i, Intercellular carbon dioxide concentration; E, Transpiration rate; and WUE, water use efficiency. Data were log-transformed (base e) before analysis to improve normality.

Notably, the responses of *L. chinensis* morphological traits to grazing in the greenhouse were markedly deceased relative to the field experiment (by 33.91%∼59.83%, **Figure 5A**). Similar to our finding in morphological traits, the responses of *P*_N_, *g*s, *C*i, and *E* of the grazed treatment in the greenhouse experiment were weakened compared to those in the field observations (by 41.67%∼92.78%, **Figure 5B**). There had positive correlations between the responses ratios in field and greenhouse both of *L. chinensis* morphological traits (*R*^2^= 0.47, *P* = 0.20) and photosynthetic traits (*R*^2^ = 0.98, *P* < 0.001, **Figure 5**).

**FIGURE 6:**
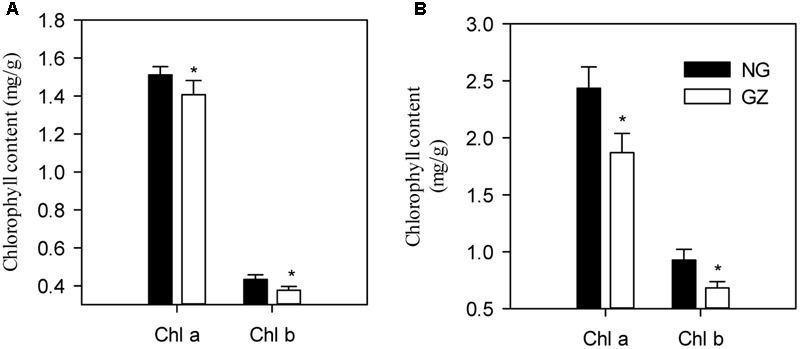
**Difference in leaf chlorophyll content for field-grown and bud-cultured *Leymus chinensis* in early growth stages under long-term overgrazing and grazing exclusion. (A)** Chlorophyll a and chlorophyll b in the field condition. **(B)** Chlorophyll a and chlorophyll b in greenhouse condition. NG, ungrazed; GZ, grazed.

**FIGURE 7:**
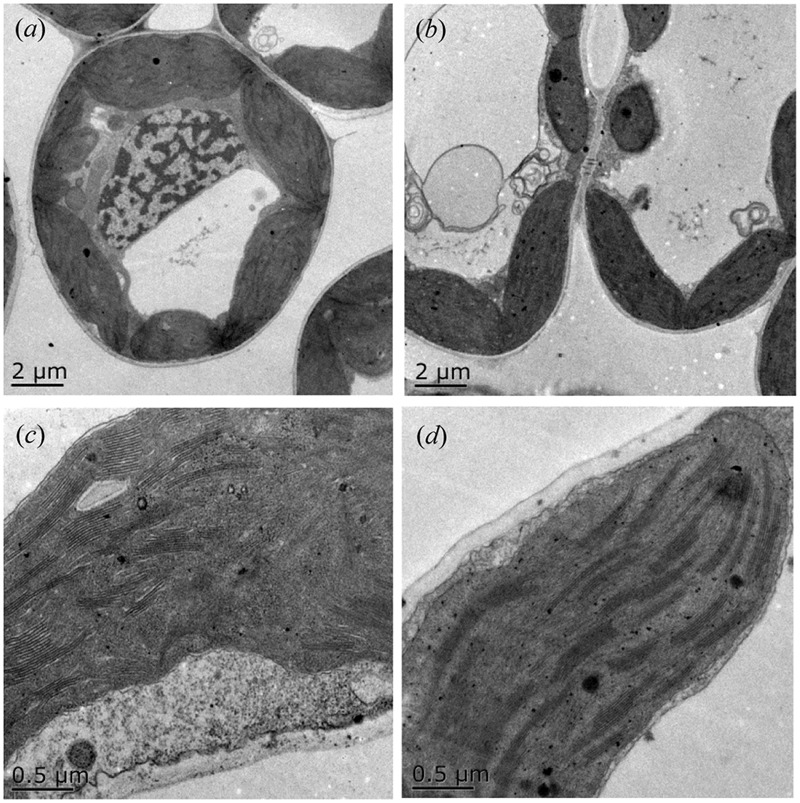
**Ultrastructure of chloroplast in ungrazed and grazed *Leymus chinensis* leaves grown from bud culture. (a,c)** The morphological characteristics of the whole cell and chloroplast of ungrazed *L. chinensis*, shown with 2 and 0.5-μm scale bars; **(b,d)** The morphological characteristics of the whole cell and chloroplast of grazed *L. chinensis*, shown with 2 and 0.5-μm scale bars.

**FIGURE 8:**
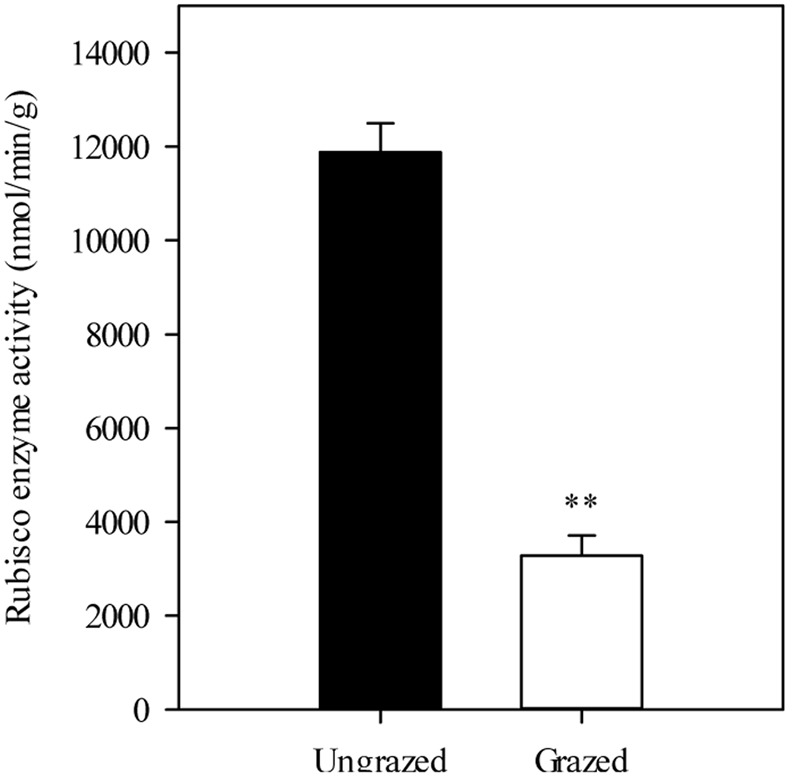
**Rubisco enzyme activities in ungrazed and grazed *Leymus chinensis* leaves grown from bud culture.** ^∗∗^*P* < 0.01.

Furthermore, we detected that long-term overgrazing significantly decreased the contents of chlorophyll a and chlorophyll b in *L. chinensis* leaves (*P* < 0.05, **Figure 6A**). After one generation of exclusion from grazing via the rhizome bud culture method, we also detected a significant difference in chlorophyll contents of leaves between dwarf and non-dwarf *L. chinensis* (*P* < 0.05, **Figure 6B**). Moreover, there were obvious changes in chloroplast ultrastructure of plants derived from grazing-stressed materials in the greenhouse experiment (**Figure 7**). Specifically, our results showed that the cells became smaller and chloroplast numbers per cell diminished, while the grana lamella concentration and osmiophilic granules increased (**Figure 7**). In addition, we found that the transgenerational memory effect of grazing-induced plant dwarf phenotypes significantly decreased leaf Rubisco enzyme activities compared with those of the non-dwarf *L. chinensis* (*P* < 0.05, **Figure 8**).

**FIGURE 9:**
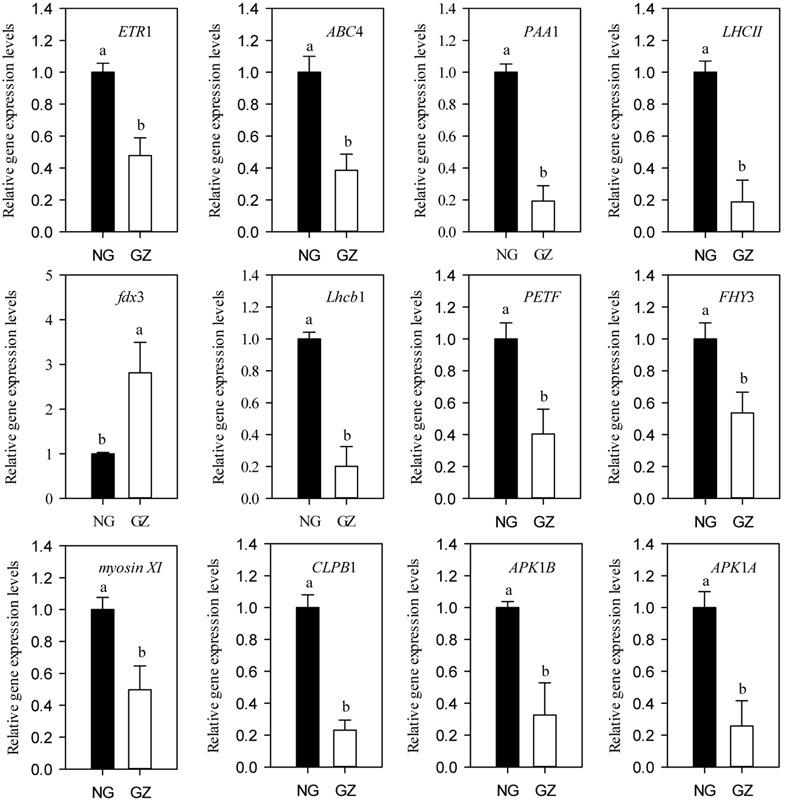
**Relative expression levels of genes related to photosynthesis from ungrazed and grazed *Leymus chinensis* leaves grown from bud culture.** Error bars represent SEs. Differing letters indicate significant differences (*P* < 0.05; *t*-test). NG, ungrazed; GZ, grazed.

To unravel the linkages between the plant morphological plasticity and leaf photosynthesis plasticity induced by livestock grazing at molecular level, we analyzed the expression patterns of a series of key genes that regulate photosynthetic efficiency, chlorophyll synthesis, and chloroplast development (Supplementary Figure S2). We found that the expression levels of the genes (except *fdx*3) in grazing-induced dwarf *L. chinensis* showed 1.86- to 5.33-fold decreases compared with those of the non-dwarf *L. chinensis* (*P* < 0.05, **Figure 9**).

## Discussion

Phenotypic plasticity, ecotypic differentiation, and ecological speciation are three different gradations of adaptation to externally ecophenotypic variation (Rausher, 2005). In many ways, the evolution of phenotypic plasticity is the most basic mode of plant species evolution and adaptation (Anderson et al., 2012; Turcotte and Levine, 2016). In this study, we initially found that field long-term overgrazing significantly decreased the individual size of *L. chinensis*. In general, phenotypic plasticity is the ability of plants to modify their phenotype in response to environmental changes. Previous studies have shown that clonal plants, such as *L. chinensis*, physiologically, morphologically, and phenologically adapt to different levels of resource availability and various kinds of environmental disturbances through phenotypic plasticity (Bai et al., 2009; Li X. et al., 2016).

Notably, our greenhouse experiments demonstrated that there was also a significant difference in plant height of *L. chinensis* between rhizome bud-cultured plants that were derived from field samples that did or did not experience grazing. Recently, an increasing number of case studies have reported that parent plants can transmit their performances in phenotypic plasticity responses as some kind of ecophenotypic variation to their offspring by seed or clonal propagation (Galloway and Etterson, 2007; Latzel et al., 2016). Our demonstration of the memory effect of the dwarf phenotype of *L. chinensis* as an adaptation to long-term overgrazing provides new evidence for clonal trans-generational memory in clonal plants. We speculate that the maintenance of phenotypic characteristic in clonal propagation may function as a grazing-avoidance for grassland plants that have adapted to grazing (N’guessan and Hartnett, 2011). Long-term overgrazing appears to induce the clonal offspring to form a phenotype that matches the grazing-avoidance phenotype in order to prepare for possible grazing by large herbivores, which was often experienced by their ancestors.

In addition, the results of this and previous studies imply that the clonal transgenerational memory of phenotype changes in the offspring may be restored in grazed grassland plants (Latzel and Klimesova, 2010b). Some restoration process research focusing on grassland degradation caused by overgrazing have reported that individual plants can return to normal conditions after several years of restoration effort (Li et al., 2008). However, the process of plasticity recovering to normal for maintained dwarf phenotype of *L. chinensis* warrants more detailed studies that utilize greenhouse experiments observed for several clonal generations. Also, more and more evidence of epigenetic inheritance in molecular biology has shown that histone variants, histone N-tail modifications, and DNA methylation affected the expression not only of within-generation phenotypic plasticity but also of transgenerational phenotypic plasticity (Iwasaki, 2015; Herman and Sultan, 2016; Wang et al., 2016). These previous empirical and theoretical studies have provided direct or indirect support for the type of phenotypic plasticity observed in *L. chinensis* in this study.

To reveal the possible reason for grazing-induced phenotypic plasticity, we detected the photosynthetic efficiency of *L. chinensis* leaves both in the field and greenhouse experiments. Long-term overgrazing significantly decreased leaf photosynthetic rates, in correspondence with the dwarf phenotype of *L. chinensis* individuals in grazed field conditions, which is consistent with previous studies across many grazing experiments conducted in semi-arid grasslands. Empirical studies focusing on the effect of grazing intensities on plant photosynthesis have indicated that defoliated plants can overcompensate for lost tissues by compensatory photosynthesis to a limited extent (Detling et al., 1979; Liu H. et al., 2016). In this study, however, long-term overgrazing restricted the compensatory effect of plant photosynthesis, which resulted in the dwarf phenotype of *L. chinensis* plants. Further evidence demonstrated that overgrazing-induced declines in photosynthesis were co-regulated by stomata size and chlorophyll content in field tests. Many studies that focused on the plant photosynthesis in response to biotic or abiotic disturbances support the results in this study to some extent (Zhao et al., 2009). Leaf photosynthetic plasticity, therefore, plays a key role in the adaptation of *L. chinensis* to long-term overgrazing through morphological plasticity.

In this research, furthermore, we found the decreases in photosynthetic functions were maintained after a generation of grazing exclusion in greenhouse experiments. These results could explain the maintenance of grazing-induced morphological plasticity of *L. chinensis* in the greenhouse experiments. Previously, we found a significant response in morphological plasticity under grazing was maintained in a hydroponic experiment designed to remove environmental variability, but there was no significant difference in *L. chinensis* individual size traits (Li et al., 2015b). In the current research, the memory effect of photosynthetic plasticity provides new information on clonal transgenerational adaptations for grazing avoidance. Moreover, there is no previous research establishing the mechanism underlying the memory effect of photosynthetic plasticity in plants that experience long-term overgrazing. We therefore posit that it is most likely associated with individual adaptive changes in the photosynthetic apparatus and hormone signaling in clonal plant species, consistent with previous studies (Whitehead et al., 2011).

The responses of morphological plasticity and photosynthetic plasticity to grazing showed that both the *L. chinensis* morphological and photosynthesis in the greenhouse were sharply deceased relative to those observed in the field tests. This suggests that grazed clones only partially maintained differences in phenotype and photosynthesis plasticity in the absence of stimuli such as livestock foraging and soil feedback. Hence, grazing-induced field morphological plasticity of plants can be divided two portions, i.e., transferable plasticity and non-transferable plasticity (von Caemmerer et al., 2004; Li et al., 2015b). This study suggests that the non-transferable plasticity is mediated by root tissue influenced by in soil bulk density, nutrient availability (Conant et al., 2001; Mcsherry and Ritchie, 2013).

It is important to detect the reasons for the clonal transgenerational effects of long-term grazed *L. chinensis.* At organelles level, we demonstrated that the adaptive changes in chloroplast development of clonal offspring of *L. chinensis* may lead to the reduction in photosynthesis directly. There were significant decreases in leaf chlorophyll contents and differences in chloroplast ultrastructure of leaves between the bud-cultured dwarf and non-dwarf *L. chinensis* plants in the greenhouse. Importantly, we detected a significant decrease in the expression of three previously putative chloroplast development genes (*CLPB1, APK1B* and *APK1A*) (Tanaka et al., 2000; Lee et al., 2007; Das et al., 2012), providing molecular evidence for developmentally limited of chloroplast in dwarf *L. chinensis*. Thus, the clonal transgenerational plasticity induced by grazing is possibly mediated by the adaptive changes in chloroplast development, which is supported by several previous studies (Reddy et al., 2002; Li et al., 2013). We posit that chloroplast development is a potential reason of dwarfism in *L. chinensis* limiting chlorophyll synthesis.

In addition to the role of the changes in chloroplast, our results imply the limitation of stomatal traits may be associated with the observed transgenerational plasticity. Decreased stomatal conductance was the primary determinant of the photosynthetic rate in bud-cultured dwarf *L. chinensis*. The down-regulated expression of gene related to stomatal regulation, for example, *ETR1*, may have contributed to the adaptive changes in the photosynthesis. Previously, some studies had proved that the *ETR1* gene played key roles in guard cell movement and stomata opening under multiple stresses (Desikan et al., 2005; Ge et al., 2015). In this research, we further speculate that smaller leaf stomata mediated by gene regulation may reduce the loss of water and improve plant fitness in grazing-induced dry habitats of semi-arid grasslands (Escalona et al., 1999; von Caemmerer et al., 2004). However, these potentially adaptive changes in stomatal traits decreased *L. chinensis* photosynthetic capacity significantly.

Moreover, we found decreasing efficiency in the light and dark reactions of photosynthesis may also be mediating the observed plasticity. It is likely that clonal transgenerational plasticity of plant photosynthetic capacity is subject to multiple photobiological processes in response to large herbivore grazing (Blankenship, 2002; Zhao et al., 2009). In addition to the role of photosynthetic organelles change, we propose that the observed phenomenon was also related to the changes in photosynthetic processes, such as light trapping and light energy transformation, and carbon assimilation. Seven of the eight assayed genes, mainly encoding pigment protein complex, showed significant decrease at transcript level between treatments, providing strong support for the process by which photosynthetic capacity decreases in *L. chinensis* clonal offspring.

In the light reaction process, we detected that there had significant decrease in the expressions of light harvesting genes (*LHCII* and *Lhcb1* for example) and photosynthetic electron transfer chain genes (*fdx3* and *PETF* for example) (Teramoto et al., 2001; Pascal et al., 2005; Grudzinski et al., 2016). In the dark reaction process, the expressions of carbon assimilation genes, such as *SR45* (Day et al., 2012), were significantly decreased in grazed *L. chinensis* clonal offspring. These results implied the photosynthetic function pathways, including the light and dark reactions, each of which may cause decreases in photosynthesis among the clonal offspring of *L. chinensis*. At enzyme level, furthermore, previous studies have indicated that Rubisco enzymes were significantly correlated with photosynthesis (Demars et al., 2016), supporting our results that the transgenerational effect was closely associated with the decrease in Rubisco enzyme activities in grazed *L. chinensis* offspring.

**FIGURE 10:**
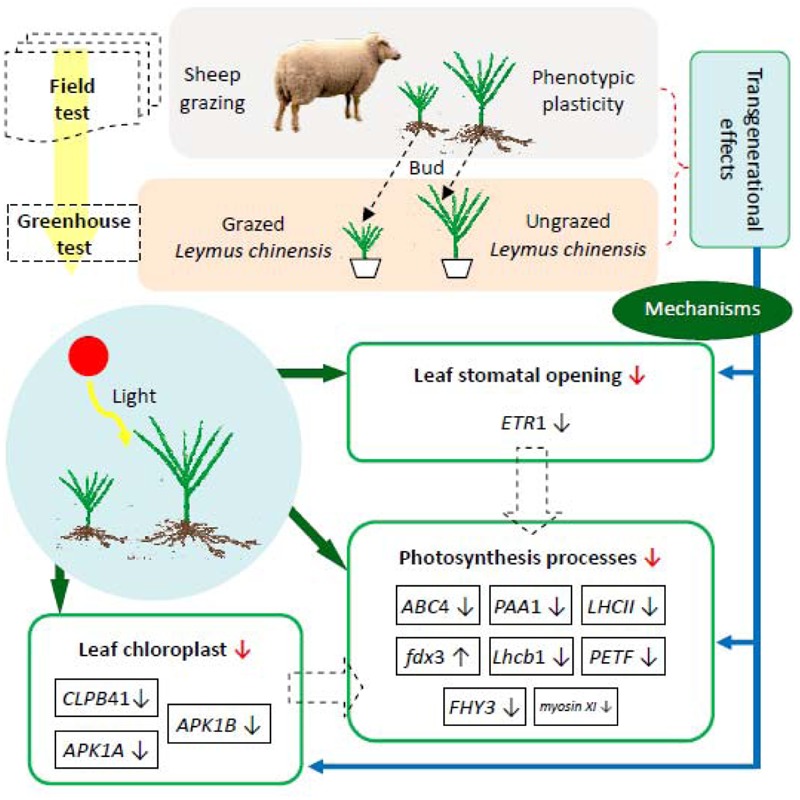
**Schematic diagram illustrating the linkages between morphological plasticity and leaf photosynthesis plasticity induced by long-term livestock overgrazing of *Leymus chinensis*. ↓** Significant decrease in photosynthesis-relevant processes; ↓ significant down-regulation of gene expression; ↑ significant up-regulation of gene expression.

In general, photosynthetic plasticity and morphological dwarfism in the clonal offspring of *L. chinensis* which experienced long-term overgrazing is an adaptation to avoid animal grazing. The field and greenhouse results characterizing morphological, physiological, and molecular responses of *L. chinensis* leaves to grazing are described in **Figure 10**, which contains schematic diagrams illustrating the linkages between the *L. chinensis* morphological plasticity and photosynthesis plasticity induced by long-term livestock overgrazing. In this figure, overgrazing-induced clonal transgenerational effect (morphological adaptation) is mediated by the adaptive changes in photosynthetic plasticity. Then under long-term grazing stress, the principal cause of the reduction in photosynthesis was attributed to the changes of structure of photosynthetic organelles (chloroplast and stomata) and function of photosynthetic processes (light and dark reactions).

## Conclusion

This study is among the first to provide strong evidence that clonal transgenerational effects are linked to adaptive changes in photosynthetic physiology in a long-term grazed plant. Both in maternal plants in the field and clonal offspring in greenhouse, leaf photosynthesis were significantly decreased by grazing of the previous generation, corresponding with the dwarf phenotype of *L. chinensis* induced by grazing disturbance. Multiple reasons underlying the transgenerational plasticity of *L. chinensis* photosynthesis were observed in this study. There were significant decreases in leaf chlorophyll contents and Rubisco enzyme activities of grazed *L. chinensis* offspring compared with offspring of ungrazed *L. chinensis*. In addition, gene expression patterns in the bud cultured dwarf *L. chinensis* exhibited a significant down-regulation of a series of key genes that regulate photosynthetic efficiency, stomata opening, and chloroplast development compared with non-dwarf *L. chinensis.*

## Author Contributions

WR, XL, XH, and NH conceived and designed the research. XL, ZL, WR and NH conducted the experiment. All authors analyzed and interpreted the data. XL and WR wrote the manuscript; all authors discussed and approved the final version.

## Conflict of Interest Statement

The authors declare that the research was conducted in the absence of any commercial or financial relationships that could be construed as a potential conflict of interest.
