# Supplementary material for: Long-Term Overgrazing-Induced Memory Decreases Photosynthesis of Clonal Offspring in a Perennial Grassland Plant
Source: Front Plant Sci. 2017 Apr 24;8:419. doi: 10.3389/fpls.2017.00419 (PMC5401901; doi:10.3389/fpls.2017.00419)
Supplement: Supplementary file 1 [file Data_Sheet_1.docx]

**Supplementary Material**

**Figure S1** The meteorological characteristics of the sampling year in the field study area.

**Figure S2** Agarose gel of total RNA extracted from different samples of *Leymus chinensis* with grazed *L. chinensis* in lanes 1–8 and ungrazed *L. chinensis* in lanes 9–16.

**Figure S3** PCR products of 13 genes. The number of 1-13 are as follows: *ETR1、ABC4、PAA1、LHCII、fdx3、Lhcb1、PETF、FHY3、myosin XI、CLPB1、APK1B、APK1A、Actin*.

**Table S1** List of primer sequences for the 13 genes subjected to qRT-PCR

**Fig.S1**

**
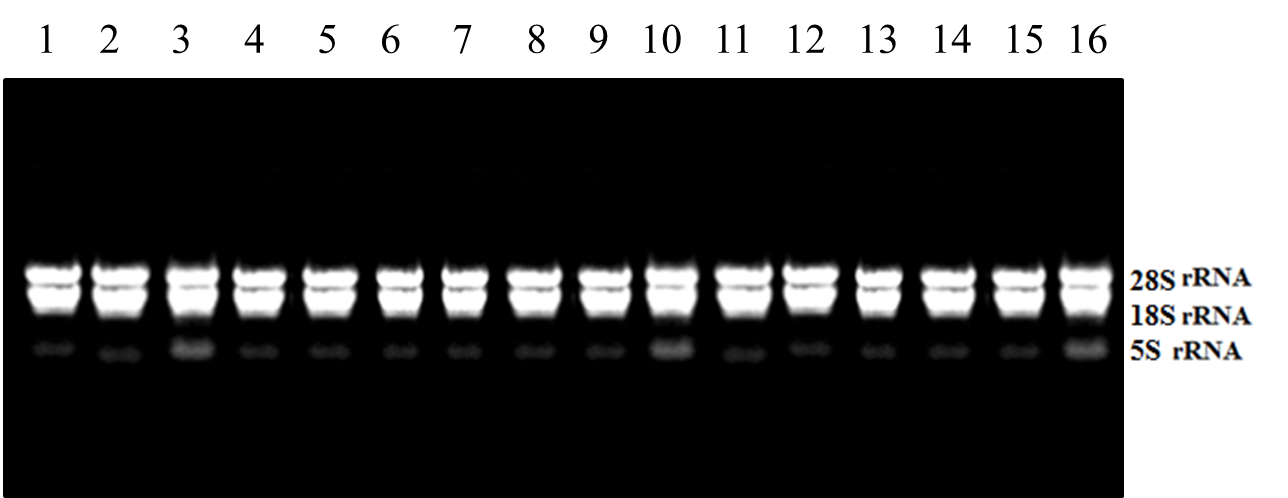
**

**Figure S2**

**
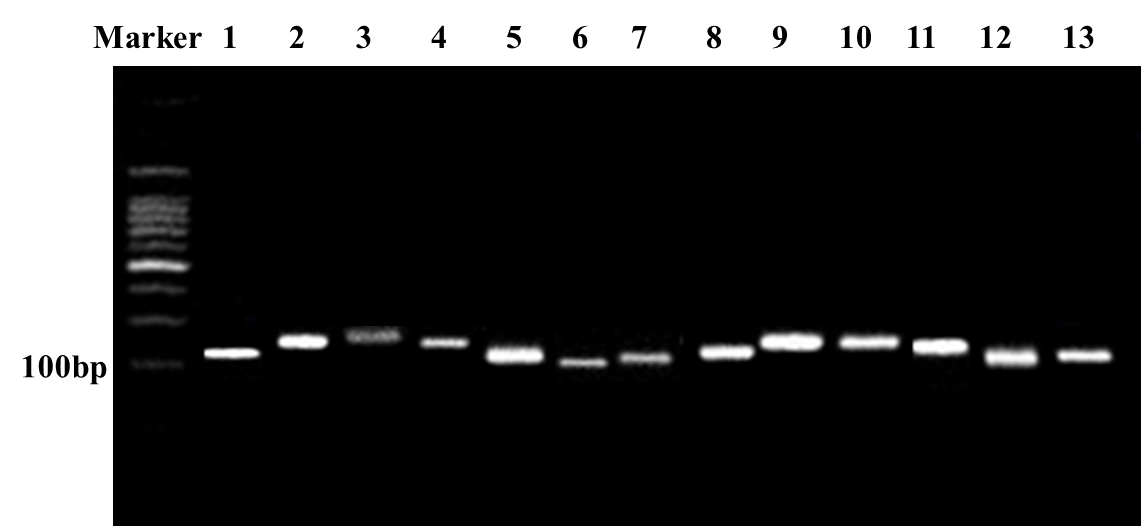
**

**Figure S3**

**Table S1**

| Gene name | Primer sequence | Product Size/bp |
| --- | --- | --- |
| *LcETR1* | TGAGGGAATGATGTTCGTTGTG | 101 |
|  | TTCGCACAGGAAGTGACCA |  |
| *LcABC4* | TCTCAAATAGCAGCAGAAGCA | 160 |
|  | AGACATCTTTCCAACAGCCC |  |
| *LcPAA1* | CCCAAGTTCCTTGTGCCA | 100 |
|  | CTGAGGTTGCTTCTGCCA |  |
| *LcLHCII* | GTACAAATCAGACCATCCCGT | 127 |
|  | GAACCTCGCTGACCATATCAC |  |
| *LcFdx3* | TCCAAGCATTGCCCAAGAG | 150 |
|  | AGACGCAAGGGAAAGGGA |  |
| *LcLhcb1* | CGCAAAGCATCCTAGCCA | 108 |
|  | GGGTAAAGTGGGTCAACAATCTC |  |
| *LcPETF* | AGGATAGTGTTGGCGCGT | 119 |
|  | TTGAGCGTGTGAACGTGG |  |
| *LcFHY3* | CTGCTGTTCCTCGAGTATTTCC | 174 |
|  | TCCCACCGTTCTTCAAATTCC |  |
| *Lcmyosin XI* | GAGTATCCTCGCCAACCAG | 179 |
|  | CTGTTTCTTCCATGCCTCTTCC |  |
| *LcCLPB1* | CAACTACGCAAGGTCGCTC | 177 |
|  | GCTCCGTCACTATCCTCTTCTC |  |
| *LcAPK1B* | TCCCACCAGAAAGACCCA | 149 |
|  | ACTTACTCCCAAACCTTGTCAG |  |
| *LcAPK1A* | ATCTTAAGGTACGCCCATCCA | 82 |
|  | TTCCTCCTTTGTCTTCGTATCC |  |
| *LcActin* | ATTGTGCTCAGTGGTGGGTCA | 136 |
|  | CCAATCCAAACACTGTACTTCCTC |  |
